# Novel Treatment to Immobilize and Use Textiles Microfibers Retained in Polymeric Filters through Their Incorporation in Composite Materials

**DOI:** 10.3390/polym14152971

**Published:** 2022-07-22

**Authors:** Francisco Belzagui, Carmen Gutiérrez-Bouzán, Fernando Carrillo-Navarrete

**Affiliations:** 1 Institut d’Investigació Tèxtil i Cooperació Industrial de Terrassa (INTEXTER), Universitat Politècnica de Catalunya, Colom 15, 08222 Terrassa, Spain; francisco.belzagui@upc.edu (F.B.); fernando.carrillo@upc.edu (F.C.-N.); 2 Department of Chemical Engineering, Escola Superior d’Enginyeries Industrial, Aeroespacial i Audiovisual de Terrassa (ESEIAAT), Universitat Politècnica de Catalunya, Colom 1, 08221 Terrassa, Spain

**Keywords:** microplastic, microfiber, treatment, textile, contamination, composites

## Abstract

Microplastics (MPs, size < 5 mm) are among the most environmentally challenging pollutants. Their continuous and cumulative inflow or generation in the environment is what makes them drastically problematic. These pollutants can come from a wide variety of sources; hence, they are potential vectors that pose extensive risks to environmental and human health. Microfibers (MFs) are one type of MPs. Among the most well-known types of MFs are those detached from textile articles from household laundering or industrial processes. Currently, there are many ways to retain the MFs detached from textile articles. However, as far we know, there are no methods of valorizing the retained MFs. As such, we propose a novel and sustainable treatment method to immobilize MFs in a polymeric matrix, turning them into a composite. To determine the mechanical properties of the expected composites, different proportions of polyester MFs were mixed with low-density polyethylene, which is the material proposed for the immobilization of MFs. The results show that the optimum manufacturing composition was 10% (*v*/*v*) polyester MFs in the polymeric matrix. This composition improved some of the tensile mechanical properties of the polymeric matrix. Once the composites are obtained, these can be used for different purposes.

## 1. Introduction

In recent years, increasing importance has been placed on reducing the pollution caused by the textile industry. As such, there are numerous studies on the emission of microplastics and microfibers caused both by industrial textile production and by the domestic laundry of garments [1,2,3].

The European Chemical Agency (ECHA) defines microplastics (MPs) as fragments of chemically modified and/or non-biodegradable polymers with a length < 5 mm [4]. These pollutants have been encountered in every assessed ecosystem; hence, they are referred to as ubiquitous [5]. Therefore, MPs have been divided into two main groups: primary MPs, which are those emitted into the environment in an MP size range, and secondary MPs, which are those generated in the environment from the fragmentation of larger plastic debris [6]. Every plastic product is a potential source of MPs. For this reason, the origin of MPs found in the environment is generally difficult to determine, except in particular cases such as cellulose acetate, which is mainly attributed to cigarette butts [7].

Microfibers (MFs) are one type of MPs and have a length-to-diameter ratio > 3 and a maximum length of 15 mm [4]. Textile MFs are among the most well-known MFs as they have been widely found in the environment. These can be byproducts of the manufacturing, usage, cleaning, or final disposal of a textile article [6]. Household laundering and textile industrial processes detach millions of MFs each year [8,9,10].

The impacts of MPs have been widely studied; for instance, ingestion across the trophic chain is now a widely known fact [11,12,13,14]. Meanwhile, some effects of MPs on organisms have been found, such as their retention in the intestinal tract and endocrine disruption [15,16,17,18]. However, such findings may be misleading, as the concentrations used in the studies differ from those usually found in the environment. Moreover, the MPs used for laboratory trials are not those that are mostly encountered, as these are MFs [19]. MPs can also behave as vectors of organisms and hydrophobic toxic compounds [20,21]. The contamination caused by MPs has also reached products for human consumption and has polluted the air [22,23,24,25,26,27,28]; hence, there are several paths for human exposure to MPs [29]. Nevertheless, the potential risks to human health are still unknown [30,31,32].

Some solutions have been proposed to retain the MFs detached from washing machines. There are in-drum accessories that can be used to reduce the generation of MFs and out-drum filters that are used to retain those generated already [33,34]. However, as far as we know, there are no existing methods to treat the retained MFs. For example, Napper et al. (2020) commented that once the filters are cleaned by collecting the MFs, they can be “thrown into the everyday household waste” [35]. Hence, the MFs could also end up in the environment. This article aims to present a novel, practical, and sustainable treatment method for the valorization of the retained MFs detached from textile garments. As such, to treat the microfibers, we propose to immobilize them into a polymeric recycled matrix from which they cannot escape. Once inside, MF-based polymeric composites can be obtained, and these composites can have various applications. The method consists of making a composite with MFs and a thermoplastic polymeric matrix, in this case, polyester (PES) and low-density polyethylene (LDPE). PES was selected as it is the most commonly used fiber in the textile industry [36,37], while LDPE was selected because of its relatively low fusion temperature. The composites were made by mixing different proportions of PES and LDPE. In this study, three different proportions of polyester microfibers (5%, 10%, and 15%, *v*/*v*) were introduced into a recycled low-density polyethylene matrix. The purpose was to gain an approximated knowledge of the highest concentration of microfibers that can be included while still being able to produce a high-quality product. The composites were subjected to tensile tests to evaluate their mechanical properties, and the morphology of the fracture’s surface was analyzed using scanning electron microscopy (SEM). The materials showed very good compatibility since some of the tensile mechanical properties of the LDPE were improved, because polyester MFs provided reinforcement of the polymer matrix. In light of these findings, we propose a novel and practical method for the valorization of textile MF pollutants.

## 2. Materials and Methods

### 2.1. Composites Manufacturing 

The composites were prepared by mixing different proportions of PES-MFs with LDPE. LDPE (ALCUDIA PE-022, Repsol-YPF, Tarragona, Spain) with a density of 0.915 g/cm^3^ was used. The mixing was carried out in a CollinW100T two-roll mixer (Dr. Collin, GmbH, Maitenbeth, Germany). The temperature in both cylinders was set at 130 °C. Once the LDPE was melted, the MFs were introduced and mixed for about 10 min to achieve sufficient mixing between the polymers. The tested compositions were 5%, 10%, and 15% (*v*/*v*) MFs in the polymeric matrix (LDPE). Higher proportions did not allow for obtaining compact composites, since more MFs would require more polymeric matrix to coat them all. Once the mixing was completed, the blend was then consolidated at 100 kN and 140 °C for 5 min in a Collin Mod. P 200E hot plate press machine (Dr. Collin GmbH, Germany, forming square plates (100 × 100 × 2.5 mm^3^)). The cooling process was carried out using cool water for refrigeration. Test samples were properly shaped according to the ASTM-D-412-98 specifications to be used in the tensile test. Plain LDPE without MFs was also treated in the same way as the filling materials to obtain suitable reference samples. 

### 2.2. Tensile Test

The tensile strength tests were carried in an Instrom 3366 (Instrom, Buckinghamshire, UK) universal machine by following the standard ASTM-D-638-14 [38]. The tensile test speed was set at 1 mm per minute. Young’s modulus, tensile strength, and elongation at maximum load were calculated using Bluehill version 2 software. Five test specimens per composite were tested and compared with pure LDPE. The mean and standard deviation were calculated and used to assess the mechanical behavior of the composites.

### 2.3. SEM Images

Scanning electron microscopy (SEM) was used to qualitatively examine the fracture surface of the broken samples from mechanical testing. By observing the environment of the MFs, it was possible to analyze the adhesion of the fibers to the matrix. Several images of every composite sample were studied. The microscope used was a Phenom G2 Pro device (FEI company, Hillsboro, OR, USA), at an accelerating voltage of 15 kV. Prior to the observations, one sample of each of the composites was prepared by coating them with a fine layer of gold–palladium alloy to increase their conductivity. 

## 3. Results

### 3.1. Composites

**Figure 1:**
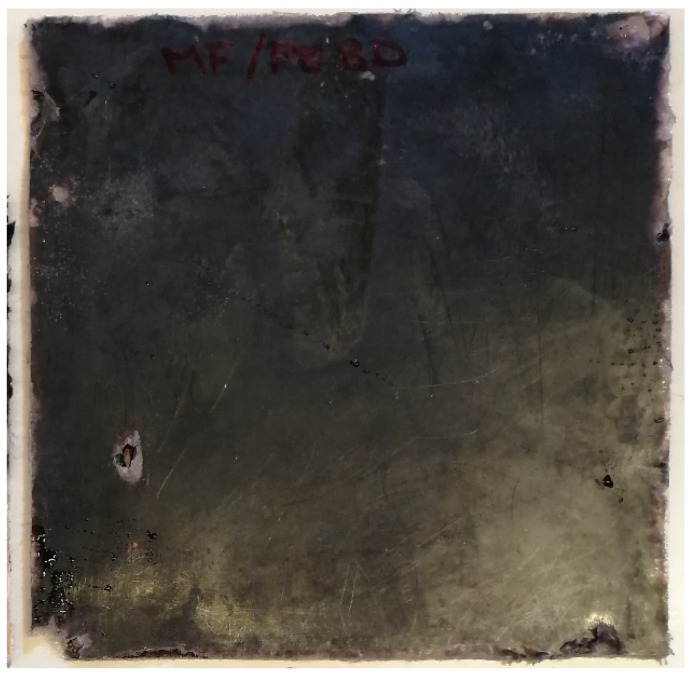
Composites made with 10% vol. of PES-MFs in an LDPE matrix. The lighter section of the photo is a visual effect of the incident light.

A picture of the upper surface of the composite with 10% MFs can be seen in Figure 1. As explained previously, different proportions were used (5%, 10%, and 15%). Visually speaking, no variation was observed between the different composites. In addition, no MFs were seen at the composites’ surface, meaning that these were completely trapped inside the polymeric matrix. This observation was considered a positive outcome, as composites with MF “limbs” did not appear on the surface. It is important to note that these composites were composed of discontinuous MFs randomly oriented in a polymer matrix. Hence, the aim of the work was not to improve the properties of a polymeric material but to get rid of the MFs that are detached from textile articles. In other words, the purpose of this work was to treat waste that is currently of concern to the scientific community.

In Figure 1, square-shaped composites are shown. However, depending on the mold used in the hot plate press machine, other shapes can be formed. The shape will only depend on the mold used in the hot plate press machine. Hence, a wide variety of products can be made, as it will only need a mold to make replications. Additionally, no novel or unconventional equipment is needed to make the composites. This treatment was developed considering the equipment commonly used in polymer recycling plants. In this sense, depending on the product that would be made, some recycling plants can introduce MFs into their pre-products without making any significant changes to their processes. 

### 3.2. Tensile Tests Outcomes

**Figure 2:**
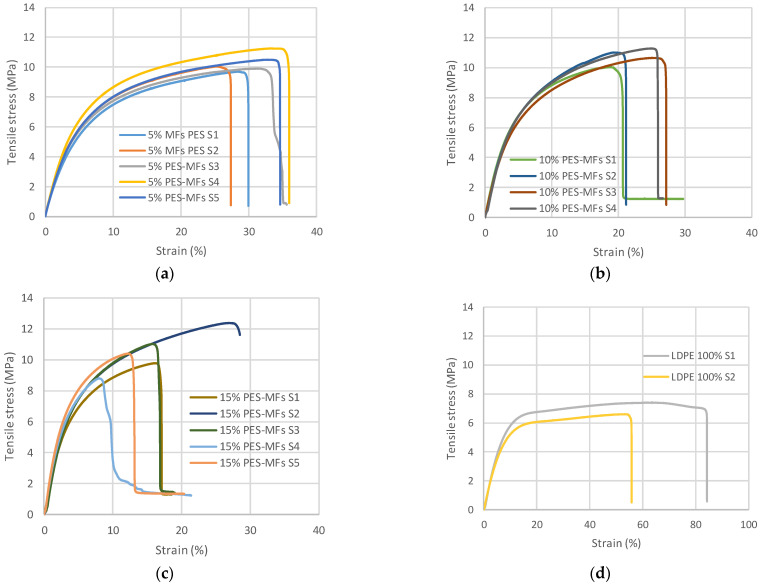
Strain (%) versus tensile stress (MPa) of composites with different compositions of polyester microfibers (PES-MFs) in the low-density polyethylene matrix (LDPE): (**a**) 5% PES-MFs; (**b**) 10% PES-MFs; (**c**) 15% PES-MFs; (**d**) pure LDPE.

Figure 2 shows the stress–strain curves for the different composite specimens and for pure LDPE. The stress is defined as the ratio of the force to the cross-sectional area of the specimen and the strain is the axial elongation. As shown in Figure 2, the tensile stress as well as the Young’s modulus improved when the MFs were inserted into the polymeric matrix. We are transforming MFs pollutants into feedstock to improve the tensile stress of the LDPE; hence, the term “garbage” could be substituted for “raw material”. Once again, it has to be emphasized that we are not dealing with fibers that could be arranged and prepared to provide a series of better properties to a given polymer, i.e., a grid to insert inside the polymer. In this case, we are working with “garbage”, MFs obtained from textile articles that are randomly inserted into the polymer matrix. 

**Figure 3:**
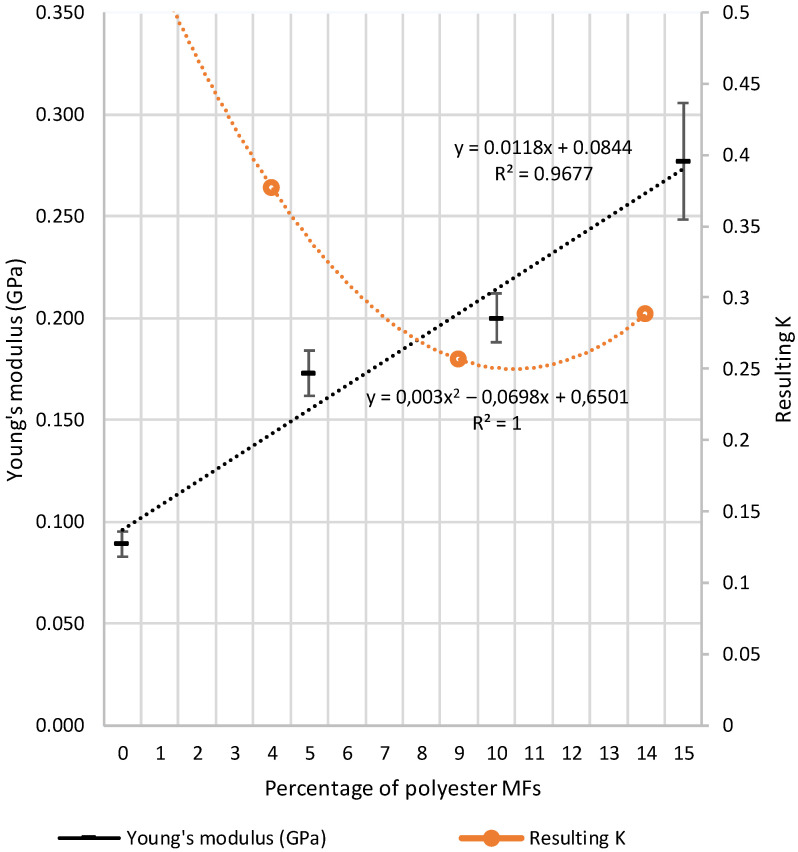
Young’s modulus and the resulting “K” for each of the composites made.

As can be seen in Figure 2, in terms of homogeneity, including 10% MFs in the polymeric matrix yielded the most preferable outcomes. This was elucidated from their standard deviation regarding the Young’s modulus and the strain: 11 for 5%, 12 for 10%, and 28 for 15% (standard deviation values for Young’s modulus, see also Figure 3). At a certain point along the line between 10% and 15% MFs, the standard deviation becomes larger, which could indicate an inflection point from which the composite begins to lose some of its homogeneity. However, depending on the application that these composites could have (i.e., non-structural applications), the 15% MF proportion of composites can be sufficiently homogeneous if the objective product is not subjected to tensile stress greater than 8 MPa.

It has to be mentioned that what is gained in tensile stress is lost in elasticity. In other words, the composite becomes more prone to rupture without a great deformation. As can be seen in Figure 2, the strain at maximum load decreases when PES-MFs content is increased. Additionally, in Figure 3, the calculated average Young’s modulus is plotted against PES-MFs content, and as can be seen, in all cases, the mean Young’s modulus increases significantly: from 89.2 ± 6.1 MPa for 100% LDPE to 172.8 ± 11.0 MPa, 200.0 ± 11.9 MPa, and 276.9 ± 28.6 MPa for composites with 5%, 10% and 15% PES, respectively. The most plausible explanation for this outcome is that the Young’s modulus of the polyester fibers is much higher than that of LDPE [39]. From our experimental data, the LDPE Young’s modulus is 9 MPa, whereas for PES, the values are between 0.92 and 10 GPa [40,41,42]. Hence, the contribution of PES microfibers causes an increase in tensile stress at maximum load compared with pure LDPE.

In addition, tensile stress is lower for the maximum load of LDPE than for composites but can withstand a longer deformation. With the obtained data, we can apply the rule of mixtures to estimate the parameter “K” [43] of Equation (1), which is a fiber efficiency parameter that gives an indication of the contribution of the MFs’ properties to the composites.

E_C_ = KE_F_V_F_ + E_M_V_M_, (1)

where:

E_C_: Young’s modulus of the composite;
E_F_: Young’s modulus of the polyester MFs (mean between 0.92 to 10 GPa, i.e., 4.64 GPa) [40,41,42];
V_F_: volume of MFs included in the matrix;
E_M_: Young’s modulus of the LDPE (0.09 GPa, experimentally obtained)
V_M_: volume of LDPE included in the matrix.

Hence, we could estimate the “K” for every measured point, and the evolution of the “K” parameter is shown in Figure 3. For each average E_C_ experimentally determined (see Figure 3 for mean E_C_ values), the value of “K” was determined from E_F_ (4.64 GPa), E_M_ (0.09 GPa), V_F_ (5, 10 or 15%) and V_M_ (95, 90 or 85%). From Figure 3, we can see that, initially, the “K” parameter decreased from 0.38 to 0.26 when the MFs’ concentration changed from 5% to 10%. However, when the MFs increased to 15%, the “K” parameter also tended to increase. These changes observed in the “K” parameter may be caused by differences in the orientation and distribution of the MFs. Hence, when the concentration of MFs is low, the fibers could be preferentially, randomly, and uniformly distributed within a specific plane expecting a “K” value of 0.375 (as mentioned in William and Rethwisch (2018) [43]). On the other hand, the increase in the MFs concentration up to 10% vol. may induce a random and uniform distribution of the fibers within the three dimensions in the space, thereby causing a reduction in the “K” parameter.

Continuing on the line between the 10% and 15%, an increase in the “K” parameter was observed, revealing a change in the composites’ behavior. Hence, with the obtained equations, we can determine the “inflection” point. As explained previously, this could be the point at which the standard deviation of the composites’ tensile stress begins to increase, corroborating the results of the tensile tests shown previously. Setting the derivative of Equation (2) equal to zero (Equation (3)), the MFs composition corresponding to the minimum “K” was found to be 11.6 %:

y = 0.003x^2^ − 0.0698x + 0.6501 (2)

dy/dx = 0 = 0.006x − 0.0698(3)

where “x” represents the percentage of MFs, and “y” represents the fiber efficiency parameter “K”. 

At a concentration of approximately 12% MFs, the composite started to become less homogeneous. This may have been caused by the MFs filling a significant space of the composites, thereby lowering the homogeneity. However, more data should be obtained, and more experimental observations should be conducted in order to arrive at a more precise conclusion. On the other hand, as can be seen in the SEM images section, the adhesion of the MFs to the matrix could be improved by introducing other types of MFs with more asperity (e.g., cotton). In real-world conditions, cotton and other MFs will be present, as the filters used to retain the MFs do not discriminate between synthetic or natural fibers.

### 3.3. SEM Images

**Figure 4:**
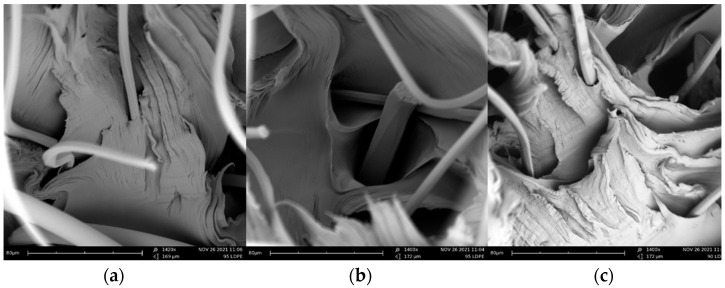
SEM images of the composites at ×1400: (**a**) and (**b**) composite of 5% PES-MFs; (**c**) composite of 10% PES-MFs.

The fiber–matrix adhesion achieved in this case can be clearly seen in Figure 4, where a type of “tunnel” is formed between the PES-MFs and the LDPE matrix. These tunnels might be formed when the LDPE contracts as a consequence of its cooling and hardening. The images reveal a lack of fiber–matrix compatibility, mainly because the PES microfibers are smooth with a negligible degree of roughness [44]. However, as previously stated, when working with real mixed materials that outflow from a washing machine, other types of MFs will also be included in the composites. In this sense, materials with more roughness will appear [45,46]: hence, mechanical adhesion is expected, thus increasing the adhesion between the MFs and the polymeric matrix. Despite the low compatibility, the composite does not necessarily have lower tensile strength, because, as stated in the previous section, the tensile strength increases when the MFs are included. Hence, to achieve a composite with improved mechanical properties, it is imperative to perform a thorough mixing before creating the final products. An important aspect to consider is that this treatment can include the MFs independently of the retaining method used (e.g., XFiltra, PlanetCare, Microplastics LINT LUV-R [35]).

### 3.4. Practical Solution

Nowadays, in Europe alone, ten million tons of LDPE are produced every year. They are used for reusable bags, trays, and food packaging, among other functions [47]. In addition, approximately 40% of the packaging waste, which commonly corresponds to a type of PE or polypropylene, is currently recycled in Europe [48]. 

Globally, there are half a million tons of MFs that are annually generated from domestic laundering [49]. The recycling of this huge amount of MFs, as a composite at 10% (*v*/*v*), requires five million tons of LDPE. In other words, half of the European production of LDPE is enough to sustainably and practically treat the detached MFs.

Our research group is currently working on some applications of the new MF composites. Some of the most promising results are found with a 10% (*v*/*v*) MF-LDPE composite used to obtain moving-bed bioreactor carriers. These carriers are being tested in biological wastewater treatment plants, and the results will be published once the project is finished.

## 4. Conclusions

PES-MFs immobilized into a polymeric recycled matrix can be transformed into a composite material. It was found that when including microfibers at a concentration of 10% in the thermoplastic polymer matrix, some mechanical properties, such as the tensile stress and Young’s modulus, improved at the expense of reducing the maximum deformation achievable. It was seen that the composites with up to 10% microfibers behaved homogeneously. A lower concentration of microfibers also worked fine, but our objective is to treat the current “fibers’ microplastic pollution from laundering”. Thus, the more microfibers that can be included, the better. Additionally, no microfibers were detached from the final composite, meaning that these were fully contained in the matrix. On the other hand, SEM images showed a low fiber–matrix compatibility due to the non-existent roughness of the PES microfibers. However, in real-world conditions, other microfibers with more roughness than that of polyester will be included, further increasing the grip between the pollutants and the recycled polymer by promoting mechanical adhesion.

## Data Availability

Not applicable.
